# Effects of Aquatic Exercise on Type 2 Diabetes Management in Adulthood: A Systematic Review and Meta-Analysis, Including Evidence on the Use of Wearable Devices

**DOI:** 10.3390/healthcare14080998

**Published:** 2026-04-10

**Authors:** Josiane Nicolle Pereira, Francisco A. Ferreira, Vinícius Costa Lima

**Affiliations:** 1 Faculty of Medicine, University of Porto, 4200-319 Porto, Portugal; josianenicollepereira.123@gmail.com; 2 Centre of Research, Education, Innovation and Intervention in Sport [CIFI2D], Faculty of Sport, University of Porto, 4200-450 Porto, Portugal; 3 Porto Biomechanics Laboratory [LABIOMEP], Faculty of Sport, University of Porto, 4200-450 Porto, Portugal; 4 RISE-Health, Faculty of Medicine, University of Porto, 4200-319 Porto, Portugal; vlima@med.up.pt

**Keywords:** diabetes mellitus, water-based exercise, land-based exercise, glycaemic control, physical fitness, technology

## Abstract

**Highlights:**

**What are the main findings?**

- Aquatic exercise significantly improves HbA1c compared with passive control conditions in adults with type 2 diabetes.
- Aquatic exercise shows similar effects to land-based exercise for glycaemic control.

**What are the implications of the main findings?**

- Aquatic exercise may be an effective and accessible alternative for individuals with barriers to land-based exercise (e.g., joint pain or obesity).
- Further high-quality trials are needed to determine optimal exercise protocols and evaluate the role of advanced wearable technologies.

**Abstract:**

**Background/Objectives**: Type 2 Diabetes Mellitus (T2DM) is a prevalent metabolic disorder associated with major cardiovascular and metabolic complications. Regular physical activity is recommended for glycaemic management, but barriers such as obesity, joint pain, and impaired mobility may limit participation in land-based exercise. Aquatic exercise may provide a feasible alternative as water buoyancy reduces joint loading while allowing aerobic and resistance training. This systematic review and meta-analysis evaluated the effects of aquatic exercise interventions on glycaemic control in adults with T2DM. **Methods**: The review followed the PRISMA 2020 guidelines. MEDLINE, Cochrane CENTRAL, Scopus, Web of Science, and IEEE Xplore databases were searched. Randomised and non-randomised longitudinal studies involving adults aged ≥35 years with T2DM participating in structured aquatic exercise programmes were eligible. The primary outcome was glycated haemoglobin (HbA1c). Risk of bias was assessed using RoB 2 and RoBANS 2, and certainty of evidence was evaluated using GRADE. Random-effects meta-analysis calculated mean differences (MDs) with 95% confidence intervals. **Results**: Eleven randomised controlled trials involving 335 participants were included. Aquatic exercise significantly reduced HbA1c compared with passive control conditions (MD = −0.76%; 95% CI −1.21 to −0.32), although heterogeneity was high. No significant differences were observed between aquatic and land-based exercise interventions. Eight studies used wearable heart-rate monitors to regulate exercise intensity. **Conclusions**: Aquatic exercise may improve glycaemic control compared with sedentary conditions and yields effects comparable to those of land-based exercise in adults with T2DM. Further high-quality trials are needed to clarify optimal exercise dose–response and evaluate more advanced wearable technologies.

## 1. Introduction

Type 2 Diabetes Mellitus (T2DM) is one of the most prevalent non-communicable diseases worldwide [1], affecting hundreds of millions of individuals and imposing a substantial clinical and economic burden on healthcare systems [1,2]. Its global prevalence continues to rise, driven by ageing populations, sedentary lifestyles, and increasing obesity rates [2]. T2DM is associated with significant microvascular and macrovascular complications, including cardiovascular disease, neuropathy, nephropathy, and musculoskeletal disorders, which collectively contribute to reduced quality of life and increased mortality [1,2].

Regular and structured physical activity constitutes an essential element of T2DM management [3]. Current guidelines from the American Diabetes Association and the World Health Organization recommend at least 150 min per week of moderate-to-vigorous aerobic exercise, combined with resistance training [3,4]. Exercise enhances insulin sensitivity, reduces glycated haemoglobin (HbA1c), improves body composition, and supports cardiovascular function [3,5,6]. However, despite strong recommendations, many individuals with T2DM face substantial barriers to engaging in land-based exercise. Obesity, joint pain, peripheral neuropathy, impaired balance, and limited mobility may compromise adherence and widen the gap between clinical guidelines and achievable patient behaviour [7,8,9].

Aquatic exercise represents a potentially valuable alternative for overcoming these limitations. The buoyancy of water reduces weight-bearing stress on joints and soft tissues, facilitating movement in individuals with obesity or musculoskeletal discomfort [10,11]. Simultaneously, water viscosity provides multidirectional resistance, allowing both aerobic and strength-based training within a low-impact environment [10,12]. These physiological properties may improve exercise tolerance, promote adherence, and enable adequate training intensity while reducing injury risk.

Aquatic exercise encompasses a heterogeneous range of modalities, including aerobic activities (e.g., swimming, deep-water running, underwater treadmill training), resistance-based exercises using aquatic equipment, and combined formats such as water aerobics or aqua fitness [13,14]. While aerobic modalities may primarily influence glycaemic regulation and cardiorespiratory fitness, resistance-based approaches may contribute to improvements in muscular strength and metabolic outcomes [15,16]. However, many interventions integrate both components, making strict categorisation challenging. This variability in modality, intensity, frequency, and duration may contribute to inconsistencies across studies and complicate the interpretation of results.

Despite these plausible physiological advantages and encouraging findings from individual trials, the evidence regarding the effectiveness and potential dose–response relationship of aquatic exercise in individuals with T2DM remains heterogeneous and fragmented [13]. Existing studies differ in intervention design, comparator groups, and outcome measures, limiting comparability and complicating the identification of consistent treatment effects. In particular, the extent to which aquatic exercise improves HbA1c has not been comprehensively quantified [15]. Furthermore, its effects on fasting blood glucose, body composition, physical conditioning, quality of life, and exercise adherence remain unclear due to inconsistencies in reporting [17].

In parallel, advances in digital health technologies have introduced wearable devices capable of monitoring physiological responses during exercise [18,19,20]. These tools may support exercise prescription and adherence by enabling real-time tracking of intensity and performance. However, their application within aquatic environments presents specific challenges, including waterproofing, signal transmission, and measurement accuracy [18,19]. Moreover, it remains unclear to what extent wearable technologies have been incorporated into aquatic exercise interventions and whether their role extends beyond exercise monitoring.

Given these uncertainties, a systematic review and meta-analysis are warranted to synthesise the available evidence and clarify the clinical relevance of aquatic exercise for adults with T2DM. This study aimed to evaluate the effects of structured aquatic exercise interventions, compared with land-based exercise or standard care, on glycaemic control in adults with T2DM, with HbA1c as the primary outcome. Secondary outcomes included fasting blood glucose, body composition, physical fitness, quality of life, and exercise adherence. Additionally, this review explored the extent to which wearable devices were used within aquatic interventions, focusing on their role in exercise monitoring rather than their independent clinical effects. We hypothesised that aquatic exercise would improve glycaemic control in adults with T2DM, particularly when compared with passive control conditions, and that its effects would be comparable to those of land-based exercise.

## 2. Materials and Methods

The present study was conducted in accordance with the Preferred Reporting Items for Systematic Review and Meta-Analyses (PRISMA) guidelines [21,22]. The protocol was registered with the International Prospective Register of Systematic Reviews (PROSPERO) on 30 October 2025, under registration number CRD420251169973 [23]. No similar protocol existed at the time of writing.

### 2.1. Search Strategy

A comprehensive literature search was conducted from database inception to December 2025. The following electronic databases were searched to identify the population, intervention, and outcomes of interest: MEDLINE (via PubMed, using MeSH terms and free-text keywords), Cochrane CENTRAL, Web of Science, Scopus, and IEEE Xplore, (Table S1).

Search terms were structured according to the PICO framework. For the population, terms included “Type 2 Diabetes Mellitus”, “Type 2 Diabetes”, “T2DM”, “Diabetes”, and “NIDDM”. For the intervention, terms included: “aquatic exercise”, “aquatic aerobics”, “aquatic sport”, “aquatic rehabilitation”, “aquatic activity”, “aquatic physical therapy”, “water-based exercise”, “water aerobics”, “water exercise”, “water sport”, “water rehabilitation”, “swimming”, “in-water exercise”, and “aquatic training”. For the outcomes, terms included: “glycated haemoglobin”, “HbA1c”, “A1c”, “Haemoglobin A1c”, “fasting blood glucose”, “blood glucose”, and “glycaemic control”.

Boolean operators (AND/OR) were used to combine population, intervention, and outcome terms. No date restrictions were applied, and only studies published in English and Portuguese were included due to feasibility and language proficiency of the review team. Reference lists of all included studies in the systematic review were also screened using the same criteria to identify additional potentially eligible studies.

### 2.2. Study Selection

Randomised and non-randomised longitudinal intervention studies evaluating the effects of structured aquatic exercise interventions, with or without wearable devices, in adults with T2DM were eligible.

The inclusion criteria applied in this study comprised (1) adults aged ≥35 years with a clinically diagnosed T2DM; (2) participants of any gender or ethnicity; (3) interventions involving structured aquatic exercise; (4) randomised or non-randomised longitudinal study designs; (5) studies reporting at least one baseline measure of glycaemic control, including HbA1c or fasting blood glucose; and (6) studies reporting that participants provided informed consent and that the study had received ethics approval.

Structured aquatic exercise was defined as any planned and supervised water-based exercise programme, including aerobic, resistance, or combined modalities. This broad definition was intentionally adopted to reflect real-world clinical practice, where aquatic exercise interventions often integrate multiple components.

The PICO framework guided the selection process. The population of interest comprised adults aged 35 years or older diagnosed with T2DM. The intervention consisted of structured aquatic programmes delivered either alone or in combination with wearable devices for monitoring or support. Comparator groups included passive control conditions (i.e., sedentary behaviour, standard care or no structured exercise) or active control conditions (e.g., land-based exercise programmes). The primary outcome was glycaemic control, assessed by HbA1c. Secondary outcomes included fasting blood glucose, body composition, measures of physical conditioning, quality of life, exercise adherence, and data derived from wearable devices when reported.

The exclusion criteria were as follows: (1) participants with Type 1 diabetes, gestational diabetes, or prediabetes only; (2) animal, in vitro, or cross-sectional studies; (3) case reports, reviews, conference abstracts, or qualitative studies; (4) participants with medical contraindications to exercise; (5) participants who had undergone recent surgery or experienced acute illness compromising participation; and (6) studies involving concurrent lifestyle interventions (e.g., combined diet and exercise programmes) in which the independent effect of aquatic exercise could not be isolated.

After identifying potentially relevant references and removing duplicates, titles and abstracts were initially screened. Full texts of potentially eligible studies were then assessed in detail, and data were extracted from those that met the inclusion criteria. Screening and selection were performed independently by two reviewers (JNP and FAF), with disagreements resolved through discussion or, if needed, by a third reviewer (VCL). Although inter-rater reliability statistics (e.g., kappa coefficient) were not calculated, this approach is consistent with standard systematic review methodology. These steps were conducted using Rayyan, a web application for systematic reviews [24].

### 2.3. Data Extraction

From each study included in the systematic review, general information about the article was extracted, including the author, year of publication, title, clinical trial registration number, country, and study aim. Data regarding the study population were also collected, including participant group, sample size, age, gender, and relevant clinical or demographic characteristics. Intervention details were recorded, including the duration, weekly frequency, session duration, exercise type (resistance, aerobic, or other), and any technology used, such as wearable devices. The primary outcome of interest was glycated haemoglobin (HbA1c). Several secondary outcomes were reported narratively, including fasting blood glucose (FBG), body composition parameters, physical conditioning measures, quality of life, exercise adherence, and wearable-device-related data.

For each study, relevant results for both primary and secondary outcomes were extracted, including baseline and post-intervention values and between-group comparisons when available. Given the expected heterogeneity in wearable device type, monitoring purpose, and reported metrics across studies, findings related to wearable technologies were planned to be synthesised narratively rather than quantitatively.

Screening of reference lists, selection of studies for inclusion, and data extraction were performed independently by two reviewers (JNP and FAF), while discrepancies in the assessment were resolved by consensus or by involving a third reviewer (VCL). Although inter-rater reliability statistics (e.g., kappa coefficient) were not calculated, this approach is consistent with standard systematic review methodology.

### 2.4. Risk of Bias Assessment

Risk of bias was assessed independently by two reviewers (JNP and VCL), with disagreements resolved by consensus or, when necessary, by consultation with a third reviewer (FAF). For RCTs, the Cochrane Risk of Bias tool version 2 (RoB 2) [25] was used. For non-randomised longitudinal studies, the Revised Risk of Bias Assessment Tool for Nonrandomised Studies of Interventions (RoBANS 2) [26] was prespecified.

The RoB 2 tool evaluates bias across five domains: bias arising from the randomisation process; bias due to deviations from intended interventions; bias due to missing outcome data; bias in the measurement of the outcome; and bias in the selection of the reported result. In accordance with the guidance provided in the RoB 2 manual [25], the overall risk of bias judgement for each outcome was determined based on the highest level of risk identified across domains. Thus, a study was classified as low risk of bias when all domains were judged to be at low risk; as having some concerns when at least one domain raised some concerns but none were rated as high risk; and as high risk of bias when at least one domain was judged to be at high risk, or when multiple domains raised some concerns that substantially reduced confidence in the result.

RoBANS 2 assesses bias in non-randomised studies across eight domains: comparability of the target group, target group selection, confounders, measurement of intervention/exposure, blinding of outcome assessors, outcome assessment, incomplete outcome data, and selective outcome reporting [26]. Each domain was rated as “low”, “unclear”, or “high” risk of bias. Since RoBANS 2 allows different approaches to assessing the overall risk of bias, three key domains (confounders, measurement of the intervention/exposure, and incomplete outcome data) were prioritised. The overall risk of bias for each study was therefore determined by the highest level of risk identified across these domains.

The overall certainty of evidence for the primary outcome (HbA1c) was evaluated using the Grading of Recommendations Assessment, Development and Evaluation (GRADE) approach, applied separately for each pre-specified subgroup comparison. Starting from high certainty, as all included studies were randomised controlled trials, the certainty of evidence was assessed across five domains: risk of bias, inconsistency, indirectness, imprecision, and publication bias. The certainty of evidence was rated as high, moderate, low, or very low, with downgrading applied when serious or very serious concerns were identified within any domain.

### 2.5. Strategy for Data Synthesis and Statistical Analyses

Data were synthesised both qualitatively and quantitatively, depending on the characteristics of the included studies. Quantitative data were pooled in a meta-analysis using mean differences (MDs) with corresponding 95% confidence intervals (CIs). Statistical heterogeneity was assessed using the I^2^ statistic and the Cochran’s Q test, with interpretation following Cochrane thresholds: 0–40% (low), 30–60% (moderate), 50–90% (substantial), and 75–100% (considerable).

When quantitative synthesis was not appropriate due to substantial heterogeneity or insufficient data, a structured narrative synthesis was conducted. Meta-regression and additional stratified analyses (e.g., by exercise dose or intensity) were not performed due to the limited number of included studies and the heterogeneity in the reporting of key variables, which would reduce the reliability and interpretability of such analyses.

### 2.6. Meta-Analysis

Where sufficient homogeneity existed across study outcomes and measurement methods, data were quantitatively pooled. The primary outcome (HbA1c, %), reported as a continuous variable, was analysed using mean differences (MD), calculated as the post-intervention difference between the treatment and control groups. This approach was selected given that all included studies were RCTs with comparable measurement scales. Where HbA1c was reported in mmol/mol, values were converted to % using the IFCC-NGSP master equation [27]: HbA1c (%) = (0.09148 × HbA1c(mmol/mol)) + 2.152.

For studies in which standard deviations (SDs) for pre- and post-intervention values were not explicitly reported, SDs were derived algebraically from available 95% confidence intervals of the change scores, following established methods for handling missing variability statistics in meta-analysis [28]. Specifically, the standard error (SE) was calculated as half the width of the 95% CI divided by the critical *t*-value for the corresponding degrees of freedom, and the SD was subsequently obtained by multiplying the SE by the square root of the sample size. For the calculation of mean differences (MDs), post-intervention means and SD were used for both aquatic and control groups. In cases where only change-from-baseline SDs were reported, they were treated as equivalent to post-intervention SDs.

A random-effects model (DerSimonian-Laird method) was used as the primary analytical method to account for expected clinical and methodological heterogeneity arising from variations in study design (e.g., intervention duration, weekly session frequency, and session duration). The between-study variance was estimated from the data using tau-squared (*τ*^2^). Individual study weights were calculated using the inverse-variance method, incorporating both within-study and between-study variance. The overall effect was tested using a Z-score, with statistical significance set at *p* < 0.05.

Individual study estimates and pooled effect sizes were presented using forest plots, accompanied by 95% confidence intervals and *p*-values. The size of each study’s marker in the forest plot was scaled proportionally to its statistical weight. Publication bias was assessed through visual inspection of funnel plots and Egger’s regression test, as the total number of included studies exceeded the recommended threshold (n ≥ 10).

To explore potential sources of heterogeneity, subgroup analyses were conducted according to comparator type (passive vs. active control conditions). These analyses were performed to enhance the clinical interpretability of the findings and should be interpreted with caution given the limited number of studies in each subgroup.

To assess the robustness of the meta-analytic findings, sensitivity analyses were conducted by sequentially excluding individual studies (leave-one-out analysis, LOO) and by restricting analyses based on study quality. The consistency of the direction and statistical significance of the pooled estimates was evaluated.

## 3. Results

**Figure 1:**
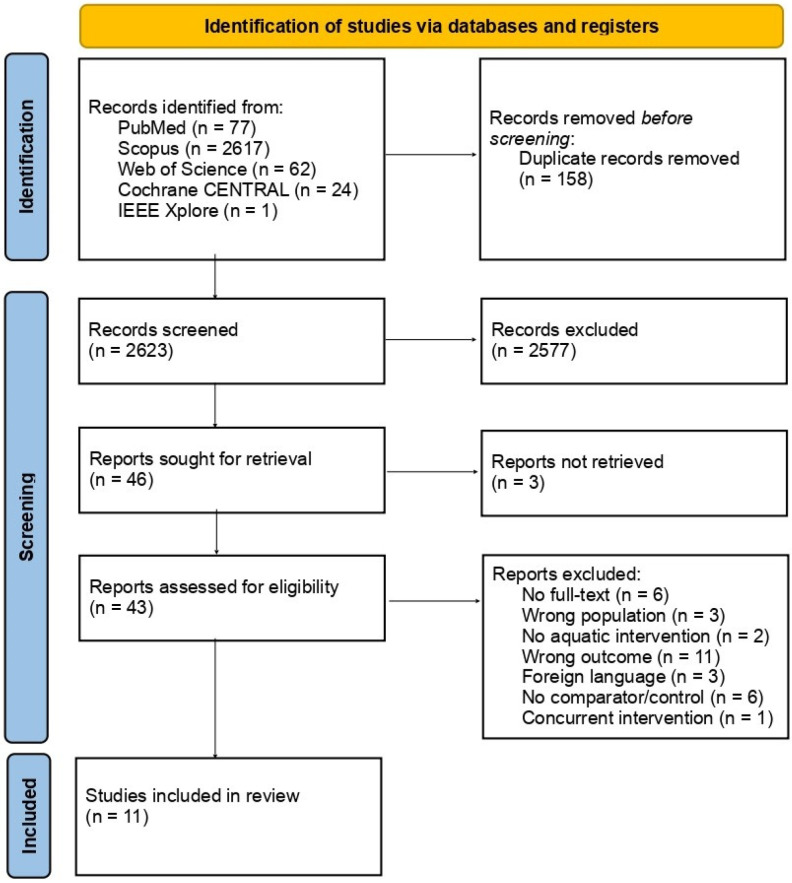
PRISMA flow diagram of the study selection process.

The study selection process is summarised in the PRISMA flow diagram (Figure 1). The database search identified 2781 records; after removing 158 duplicates, 2623 were screened based on title and abstract. Following this screening process, 43 full-text articles were assessed for eligibility. Of these, 32 studies were excluded for the following reasons: no full text available (n = 6), wrong population (n = 3), no aquatic intervention (n = 2), wrong outcome (n = 11), foreign language (n = 3), absence of a comparator or control group (n = 6), and concurrent intervention (n = 1). Examples of excluded studies included one study lacking a comparator or control group [29] and a pilot study [30].

### 3.1. Characteristics of Included Studies

Although non-randomised studies were eligible under the predefined criteria, only 11 RCTs met the final inclusion criteria and were included in the qualitative synthesis. A summary of study characteristics is presented in Table 1 (and detailed in Table S2). The 11 included studies were published between 2012 [31,32] and 2023 [33,34,35] and were conducted in several countries, including Thailand [32,34,36,37], Australia [15], Iran [35,38], Sweden [31], Brazil [39], and the United States [40].

A total of approximately 335 participants with T2DM were included across all studies. Participants were predominantly middle-aged or older adults, with several studies specifically targeting elderly populations or individuals with comorbidities such as heart failure [31] or diabetic neuropathy [38]. The intervention duration ranged from 8 [15,31,33,38] to 12 [32,34,35,36,37,39,40] weeks in all trials, with training frequency typically between 2 and 3 sessions per week and session duration generally ranging from 30 to 60 min. Water-based exercise interventions included aerobic (e.g., aqua-aerobic exercise, deep-water running, underwater treadmill training, Nordic walking performed in water [15,31,33,34,38,40]), resistance (e.g., hydrotherapy resistance [33,38]) or combined modalities [35]. Comparator groups included land-based exercise programmes, standard care, or no-exercise control conditions. All included studies assessed glycated haemoglobin (HbA1c) as a primary or secondary outcome. Several studies also reported fasting blood glucose (FBG), body composition parameters (e.g., body mass index and body fat percentage), cardiorespiratory fitness, and muscle strength. Overall, substantial variability was observed across studies in terms of exercise modality (aerobic, resistance, or combined), intervention duration, session frequency, and intensity.

Several studies reported reductions in HbA1c levels following aquatic exercise interventions compared with control groups, particularly those receiving standard care or no exercise. Improvements in glycaemic markers were observed in several studies following aquatic exercise interventions [32,34,35,40]. In contrast, studies comparing aquatic exercise with land-based exercise programmes generally reported comparable effects between interventions rather than clear superiority of aquatic training, as observed in studies [37,39]. Some studies reported small or non-significant changes in HbA1c levels, reflecting variability in intervention characteristics such as exercise modality, intensity, and programme duration. Overall, the findings of the individual trials suggest that aquatic exercise may improve glycaemic control, although the magnitude and consistency of these effects differed across studies.

This clinical and methodological heterogeneity may have contributed to differences in reported outcomes and should be considered when interpreting the results of the meta-analysis. Due to substantial heterogeneity in the definition, measurement, and reporting across studies, the secondary outcomes were synthesised narratively rather than quantitatively.

### 3.2. Risk of Bias Assessment

Although non-randomised studies were prespecified as eligible, no non-randomised studies met the final inclusion criteria. Therefore, RoBANS 2 was not applied in the final analysis. Since only RCTs were included, the risk of bias of the included 11 studies was assessed using the RoB 2 tool for the outcome HbA1c. Overall, four studies were judged to have a low risk of bias [33,38,39,40], six studies were rated as having some concerns [31,32,34,35,36,37], and one study was considered to have a high risk of bias [15]. Regarding bias arising from the randomisation process, five studies were rated as low risk, five presented some concerns, and one was judged as high risk. The study was classified as having a high risk of bias due to a lack of random allocation in the final study design.

**Figure 2:**
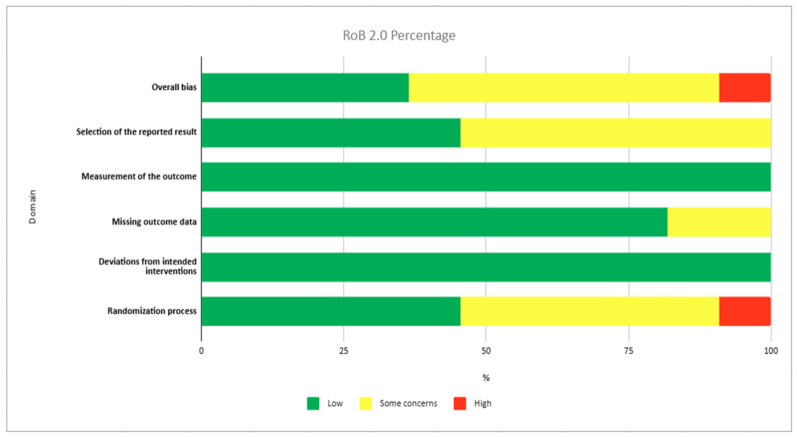
Risk of bias assessment of the included randomised controlled trials using the RoB 2 tool for the outcome HbA1c for each domain.

All included studies were judged to have low risk of bias for deviations from intended interventions (Figure 2). Similarly, all studies were considered to have a low risk of bias in the measurement of the outcome, as HbA1c is an objective laboratory-based measure, reducing the likelihood of measurement bias. In the domain of missing outcome data, nine studies were rated low-risk, while two studies raised some concerns, mainly due to incomplete attrition reporting. For the selection of the reported results, five studies were rated low-risk, and six were judged to have some concerns, particularly due to the absence of publicly available protocols or trial registration.

### 3.3. Meta-Analysis of HbA1c

For the meta-analysis, ten studies were included in the quantitative synthesis, as one study [38] did not report the necessary scalar metrics required for analysis. A random-effects model was used to pool the results, given the expected clinical and methodological heterogeneity among the included trials. The overall meta-analysis showed a non-significant reduction in HbA1c following aquatic exercise interventions compared with the control conditions (MD = −0.44; 95% CI −0.97 to 0.09; Z = 1.63; *p* = 0.10), but considerable statistical heterogeneity was observed across studies (Chi^2^ = 156.09, df = 9, *p* < 0.001; I^2^ = 94%; *τ*^2^ = 0.58).

#### Subgroup Analysis by Comparator Type

To further explore potential sources of heterogeneity, subgroup analyses were conducted according to comparator type (passive control, n = 7; active control, n = 3). The passive control subgroup included studies [15,31,32,33,34,35,40], while the active control subgroup included studies [36,37,39]. In studies comparing aquatic exercise with passive control conditions, the meta-analysis demonstrated a significant reduction in HbA1c levels following aquatic exercise interventions (MD = −0.76; 95% CI −1.21 to −0.32; Z = 3.38; *p* = 0.001). Considerable heterogeneity was observed among these studies (Chi^2^ = 56.27, df = 6, *p* < 0.001; I^2^ = 89%; *τ*^2^ = 0.26).

In contrast, studies comparing aquatic exercise with active control conditions showed no statistically significant difference between interventions (MD = 0.21; 95% CI −0.09 to 0.50; Z = 1.38; *p* = 0.167). No statistical heterogeneity was observed among these studies (Chi^2^ = 1.96, df = 2, *p* = 0.38; I^2^ = 0%; *τ*^2^ = 0.00), indicating high consistency across trials. These findings suggest that the observed effects may depend on the type of comparator, although the small number of studies and variability across interventions should be considered when interpreting these subgroup results.

The summary statistics and effect estimates for HbA1c across the included studies are presented in Table S3.

### 3.4. Sensitivity Analysis

The analysis on sensitivity through LOO indicated that the overall model (n = 10) was moderately influenced by individual studies. While the direction of the effect consistently favoured aquatic exercise, statistical significance was dependent on the inclusion of [39] and [33]. Specifically, the omission of [39] resulted in a shift to statistical significance (*p* = 0.008), indicating that this study exerted a conservative influence on the overall model.

Crucially, these two studies [33,39] represent the lowest risk of bias in the current evidence base; therefore, these findings should be interpreted cautiously, as the exclusion of higher-quality studies may introduce bias into the pooled estimate. Conversely, the removal of studies with a higher risk of bias, such as [15], did not significantly alter the pooled results (MD = −0.50, *p* = 0.074), further suggesting that the most reliable evidence supports a trend toward a beneficial effect, even if the global model remains sensitive to the high-quality data provided by [33,39].

### 3.5. Use of Wearable Technologies

Eight of the eleven studies reported the use of technological devices to monitor exercise intensity during aquatic training sessions [32,34,35,36,37,38,39,40]. Heart rate (HR) monitoring represented the most common method used to control exercise intensity and ensure adherence to the prescribed training load. In particular, waterproof HR monitors manufactured by Polar^®^ were frequently used across studies, including the Polar FT7, Polar Team 2 Pro, Polar RSX300, and Polar H10 (Polar, Oulu, Finland). These devices enabled continuous monitoring of cardiovascular responses during aquatic exercise sessions and allowed researchers to standardise exercise intensity across participants. However, no studies specifically evaluated the independent effect of wearable devices on glycaemic outcomes. Therefore, the available evidence primarily reflects their use as tools for monitoring and standardising exercise intensity rather than as intervention components influencing clinical outcomes.

### 3.6. Certainty of Evidence (GRADE)

The certainty of evidence was assessed separately for each pre-specified subgroup using the GRADE approach (Table S4). For the comparison of aquatic exercise versus passive control conditions, the certainty of evidence was rated as low. Starting from high certainty, as all included studies were RCTs, the evidence was downgraded by one level due to risk of bias, as the majority of contributing studies were rated as having some concerns under the RoB 2 tool, primarily in the domains of the randomisation process and selection of reported results, and one study [15] was rated as high risk.

A further downgrade was applied for inconsistency. Considerable statistical heterogeneity was observed (I^2^ = 88%; Chi^2^ = 56.27; df = 6; *p* < 0.001; *τ*^2^ = 0.25), and the direction of the post-intervention between-group effect was not fully consistent across the seven contributing studies, with four studies favouring aquatic exercise and three favouring the control condition. Therefore, confidence in the pooled estimate remains limited despite its statistical significance.

No downgrading was applied for indirectness, as the population (adults with T2DM), intervention (structured aquatic exercise), comparators (standard care or no structured exercise), and outcomes (HbA1c) were directly relevant to the clinical question. No downgrading was applied for imprecision, as the confidence interval did not cross the null and remained within a range considered potentially clinically relevant for HbA1c change (MD = −0.76%; 95% CI −1.21 to −0.32; *p* = 0.001). Publication bias was assessed through visual inspection of the funnel plot, which appeared symmetrical; however, this assessment should be interpreted with caution given the limited number of included studies.

For the comparison of aquatic exercise versus the active control conditions, the certainty of evidence was also rated as low. Starting from high certainty, the evidence was downgraded by one level due to risk of bias, as most contributing studies were rated as having some concerns under the RoB 2 tool, with concerns arising primarily from the randomisation process and selective outcome reporting. A further downgrade was applied for the imprecision, as the pooled estimate was based on only three studies with a combined sample of approximately 53 participants, and the confidence interval crossed the null (95% CI −0.09 to 0.50; *p* = 0.167), indicating uncertainty compatible with both a small benefit and no meaningful effect.

No downgrading was applied for inconsistency, as no statistical heterogeneity was observed across the three contributing studies (I^2^ = 0%; Chi^2^ = 1.96; df = 2; *p* = 0.38), indicating consistent findings. No downgrading was applied for indirectness, as the populations, interventions, comparators, and outcomes were directly relevant to the clinical question. Publication bias could not be formally assessed due to the insufficient number of studies in this subgroup.

**Figure 3:**
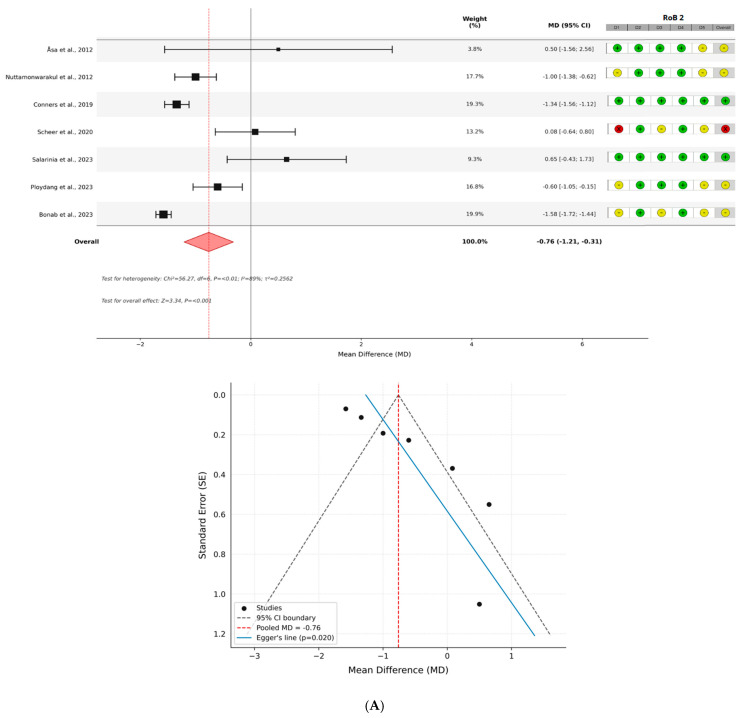

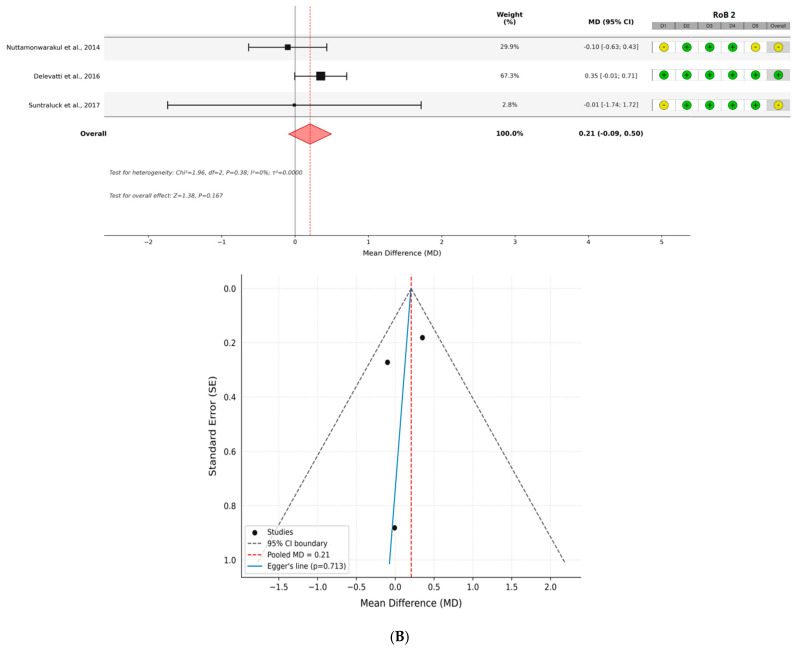
Forest and Funnel Plots for effects of aquatic vs. (**A**) passive [15,31,32,33,34,35,40] and (**B**) active control groups [36,37,39] for HbA1c, and RoB 2 judgements. Forest plots’ grey vertical line indicates no effect and the dashed red line indicates the pooled mean difference derived from the random-effects model, while red diamond represents the overall pooled effect. RoB 2 judgments reflect low (+), unclear (−), or high (×) for each domain—bias arising from (D1) randomisation process, (D2) deviations from intended interventions, (D3) missing outcome data, (D4) measurement of the outcome, (D5) selection of the reported result and overall.

The results of the meta-analysis are illustrated in the forest plots (Figure 3).

## 4. Discussion

This systematic review and meta-analysis synthesised evidence from 11 randomised controlled trials investigating the effects of aquatic exercise on glycaemic control in adults with T2DM. The overall meta-analysis did not demonstrate a statistically significant reduction in HbA1c when aquatic exercise was compared with control conditions. However, subgroup analyses suggested that aquatic exercise is associated with improvements in HbA1c when compared with passive control conditions while demonstrating comparable effects to land-based exercise interventions. These findings suggest that aquatic exercise may represent a feasible alternative strategy for improving glycaemic outcomes, although the magnitude and consistency of these effects varied according to intervention characteristics and comparator type.

The included studies comprised approximately 335 participants, most of whom were middle-aged or older adults. According to current American Diabetes Association (ADA) guidelines, screening for diabetes in asymptomatic adults should begin at age 35 years, highlighting the increased risk of T2DM from this stage of adulthood onwards [41]. This age threshold corresponds to a life stage characterised by the progressive accumulation of metabolic risk factors, including sedentary behaviour, weight gain, and declining insulin sensitivity [42]. Furthermore, adulthood is associated with lifestyle and physiological changes that may contribute to the onset and progression of T2DM, such as reduced physical activity levels and increased cardiometabolic risk [42]. Restricting the population to adults aged 35 years and older therefore enhanced the clinical relevance of the review and ensured greater population homogeneity.

A key finding of this review is the considerable heterogeneity observed across included trials, which substantially limits the interpretability of the pooled estimates. Variability in exercise modality (aerobic, resistance, or combined), intervention duration, session frequency, and intensity likely contributed to the inconsistency in outcomes. These factors may influence physiological responses to exercise, including insulin sensitivity, glucose uptake, and overall metabolic adaptation [43], thereby affecting the magnitude of HbA1c changes across studies.

When aquatic exercise was compared with passive control conditions, a statistically significant reduction in HbA1c was observed, since the magnitude of reduction (MD = −0.76%) falls within a range often considered clinically relevant. However, the substantial heterogeneity and inconsistency across studies, together with the low certainty of evidence, suggests a limit of confidence since the dose–response is not clear due to heterogeneity. In contrast, no statistically significant differences were observed when aquatic exercise was compared with active control conditions, including land-based exercise programmes. This suggests that aquatic exercise should be considered as a viable alternative rather than a superior modality. This finding may have clinical relevance, particularly for individuals who are unable or unwilling to engage in conventional land-based exercise.

Although several studies assessing diabetes control have reported alternative glycaemic indicators, such as FBG measured through capillary blood samples, these measures are more susceptible to short-term physiological fluctuations and may introduce variability when evaluating the longitudinal effects of exercise interventions [44,45]. For example, some studies examining aquatic exercise interventions have relied primarily on FBG as the main outcome measure [44]. Although FBG and HbA1c share a longitudinal association, variations in FBG may not fully capture sustained metabolic adaptations to exercise interventions [46]. Furthermore, additional factors such as the use of insulin therapy (which was not consistently controlled across the studies included in the present review) may further influence these outcomes and contribute to variability in glycaemic markers [45]. In contrast, HbA1c reflects the average blood glucose concentration over approximately two to three months and is therefore considered a more stable and clinically relevant indicator of long-term glycaemic control. For this reason, the present study prioritised HbA1c as the primary outcome measure to provide a more robust assessment of the sustained effects of aquatic exercise interventions in individuals with T2DM. Nevertheless, monitoring glycaemic-related variables during exercise may still be beneficial, as it could facilitate better control of training intensity and allow clearer assessment of the acute metabolic responses to individual exercise sessions [47].

The findings of the present review are consistent with previous evidence demonstrating the beneficial role of regular exercise in the management of T2DM. Structured interventions are widely recognised for improving insulin sensitivity, glycaemic control, and overall cardiometabolic health. Previous meta-analyses examining land-based exercise interventions in individuals with T2DM have reported HbA1c reductions typically ranging between approximately 0.5% and 0.7% [36,37,39]. The magnitude of effect observed in the present review is therefore broadly consistent with the metabolic improvements reported for traditional exercise programmes. Adopting a holistic view, the sensitivity analysis confirmed that the overall findings remained robust to the exclusion of studies with a high risk of bias [48]. Notably, the statistical significance of the model was only altered when studies with the lowest risk of bias were omitted, suggesting that the current evidence is primarily driven by the highest-quality data available.

From a physiological perspective, aquatic exercise offers several potential advantages. The buoyancy of water reduces joint loading and may facilitate participation in individuals with obesity, musculoskeletal limitations, or reduced mobility [10,11,49]. At the same time, water viscosity generates continuous multidirectional resistance during movement, which may simultaneously stimulate aerobic metabolism and muscular activation. However, the extent to which these physiological mechanisms translate into consistent clinical improvements may depend on key intervention characteristics, including exercise intensity, duration, frequency, and modality (aerobic, resistance, or combined). Variability in these factors across studies may partly explain the heterogeneity observed in the present meta-analysis and the inconsistency in glycaemic outcomes.

Adherence represents an important determinant of long-term exercise effectiveness in individuals with T2DM. Aquatic exercise may facilitate participation by reducing discomfort associated with weight-bearing activities and providing a supportive exercise environment. However, adherence was not systematically assessed across the included studies, and therefore these considerations should be interpreted as theoretical rather than evidence-based findings.

Low certainty of evidence was observed for the effect of aquatic exercise on HbA1c in both subgroup comparisons. For the passive control comparison, two domains contributed to downgrading: risk of bias, driven by concerns in the randomisation process and selective outcome reporting across most contributing studies, as well as the high risk-of-bias classification of one study [15], and inconsistency, reflecting both the considerable statistical heterogeneity (I^2^ = 88%; *τ*^2^ = 0.25) and the absence of a consistent direction of effect across contributing studies, with four studies favouring aquatic exercise and three favouring the control condition. Despite a statistically significant pooled estimate (MD = −0.76%; 95% CI −1.21 to −0.32; *p* = 0.001), the substantial between-study variance and the inconsistency in individual study findings limit confidence in the summary effect. These methodological concerns are consistent with the limitations observed across the included studies and should be considered when interpreting the pooled estimates.

For the active control comparison, downgrading was driven by risk of bias and imprecision, the latter reflecting the small number of studies and participants and a confidence interval that crossed the null. The absence of statistical heterogeneity (I^2^ = 0%) in this subgroup suggests that aquatic and land-based exercise may produce comparably similar effects on HbA1c. However, the evidence base remains too limited to draw definitive conclusions. These findings underscore the need for future well-designed RCTs with larger sample sizes, standardised intervention protocols, and prospective registration to strengthen the certainty of evidence in this field.

Beyond glycaemic outcomes, another relevant observation from this review concerns the use of wearable technologies during aquatic interventions. Most of the included studies used waterproof heart rate monitors, particularly devices manufactured by Polar^®^ (Finland), to monitor training intensity and ensure adherence to the prescribed exercise load. In this context, wearable devices were primarily employed as tools for exercise monitoring and intervention standardisation rather than as independent components influencing glycaemic outcomes. Although wearable devices are within the scope of this review, the identified evidence was primarily limited to the operational use of waterproof heart rate monitors for exercise intensity control during aquatic interventions. Therefore, the contribution of wearable technologies in the present review should be interpreted mainly as evidence of the feasibility of implementation and monitoring, rather than as evidence of an independent effect of these devices on glycaemic outcomes. This distinction is important, as none of the included studies was specifically designed to compare different wearable technologies or to determine whether device-supported monitoring improved metabolic outcomes beyond the effects of the exercise intervention itself.

Recent clinical recommendations increasingly support the use of continuous glucose monitoring systems for the management of diabetes in daily life and during physical activity [50]. Some modern devices also present waterproof characteristics that may allow their use during aquatic exercise. However, none of the studies included in the present review reported the use of continuous glucose monitoring systems during aquatic exercise interventions. Therefore, the available evidence on wearable devices in this review primarily relates to their practical role in intervention delivery and monitoring, rather than to a distinct clinical effect of wearable technology on glycaemic outcomes.

Several limitations related to the included studies should be considered when interpreting the findings of this review. First, the high degree of heterogeneity across studies limits the comparability of results and the strength of the conclusions. Second, the relatively small number of included studies, particularly in subgroup analyses, reduces statistical power. Third, variability in intervention characteristics and outcome reporting complicates interpretation. Fourth, the inclusion of only English and Portuguese language studies may have introduced language bias. Finally, the lack of standardised reporting on wearable device use limits the ability to draw conclusions regarding their potential role.

Despite these limitations, the findings of this review have potential clinical implications. Aquatic exercise may represent a valuable alternative for individuals with T2DM who face barriers to traditional land-based exercise, such as joint pain, obesity, or impaired mobility. The comparable effects observed between aquatic and land-based exercise suggest that aquatic exercise intervention programmes may provide an inclusive option for promoting physical activity and supporting glycaemic management. Future research should focus on well-designed, adequately powered randomised controlled trials with standardised aquatic exercise protocols and longer follow-up periods. Greater attention should also be given to the role of wearable technologies, particularly in terms of their potential to support exercise monitoring and optimise training intensity in aquatic environments.

## 5. Conclusions

Aquatic exercise may represent a feasible and potentially beneficial alternative for improving glycaemic control in adults with type 2 diabetes mellitus, particularly when compared with passive control conditions. However, the overall meta-analysis did not demonstrate a statistically significant effect, and no advantage was observed when compared with land-based exercise.

The considerable heterogeneity across studies, together with the relatively small sample sizes and low certainty of evidence, limits the strength and generalisability of these findings. Therefore, the results should be interpreted with caution.

Further high-quality, adequately powered randomised controlled trials with larger sample sizes, standardised intervention protocols, and longer follow-up periods are required to better establish the clinical effectiveness of aquatic exercise in this population.

## Figures and Tables

**Table 1:** Characteristics of included studies.

Author, Year (Country)	n (n per sex) Age	Aquatic Intervention	Comparator Group	Duration, Frequency	Technologies	Main HbA1c Finding: - Within Aquatic (Pre-Post) - Between Groups (Post)
Åsa et al., 2012 [31] (Sweden)	20 (4 F|16 M) 65.8 ± 5.8 y	10; Aerobic	10; Standard care (passive)	8 weeks, 3×/week	-	↘ =
Nuttamonwarakul et al., 2012 [32] (Thailand)	40 (N.R) >60 y	20; Aerobic	20; Standard care (passive)	12 weeks, 3×/week	Heart rate monitor (Polar Team 2 Pro, Oulu, Finland)	↘ ↓
Nuttamonwarakul et al., 2014 [36] (Thailand)	19 (19 F|0 M) 60–70 y	10; Aerobic	9; Land-based exercise (active)	12 weeks, 3×/week	Heart rate monitor (Polar Team 2 Pro, Oulu, Finland)	↘ =
Delevatti et al., 2016 [39] (Brazil)	21(N.R) 54.2 ± 8.3 y	11; Aerobic	10; Land-based exercise (active)	12 weeks, 3×/week	Heart rate monitor (Polar, RS300X, Oulu, Finland)	↘ ↓
Suntraluck et al., 2017 [37] (Thailand)	29 (N.R) 60–75 y	15; Aerobic	14; Land-based exercise (active)	12 weeks, 3×/week	Heart rate monitor (Polar FT7, Oulu, Finland)	↘ =
Conners et al., 2019 [40] (USA)	26 (16 F|10 M) 58.0 ± 5.0 y	13; Aerobic	13; Standard care (passive)	12 weeks, 3×/week	Heart rate monitor (Polar, Oulu, Finland)	↘ ↓
Shourabi et al., 2020 [38] (Iran)	39 (N.R) 49.8 ± 2.3 y	10; Resistance	9; Standard care (passive)	8 weeks, 3×/week	Heart rate monitor (Polar, Oulu, Finland)	↘ N.R
Scheer et al., 2020 [15] (Australia)	27 (1 2F|15 M) 60.9 ± 9.6 y	13; Aerobic	14; Standard care (passive)	8 weeks, 3×/week	-	→ =
Salarinia et al., 2023 [33] (Iran)	48 (48 F|0 M) 41.5 ± 2.7 y	10; Resistance	10; Standard care (passive)	8 weeks, 3×/week	-	↘ ↑
Ploydang et al., 2023 [34] (Thailand)	33 (21 F|12 M) 68.9 ± 3.7 y	16; Aerobic	17; Standard care (passive)	12 weeks, 3×/week	Heart rate monitor (Polar H10, Oulu, Finland)	↘ ↓
Bonab et al., 2023 [35] (Iran)	60 (60 F|0 M) 60 (60 F| 0M)	20; Aerobic and Resistance	20; Standard care (passive)	12 weeks, 3×/week	Heart rate monitor (Polar, Oulu, Finland)	↘ N.R

Note: all included studies were RCTs. Abbreviations: F: female; M: male; N.R: not reported; →: do not change; ↘: reduction; =: aquatic group similar to control; ↓: aquatic group lower than control; ↑: aquatic group higher than control.

## Data Availability

No new data were created or analyzed in this study.

## Supplementary Materials

The following supporting information can be downloaded at: https://www.mdpi.com/article/10.3390/healthcare14080998/s1, File S1: PRISMA_2020_checklist; Table S1: Search queries used in different databases; Table S2: Characteristics of included studies; Table S3: Summary of HbA1c outcomes across included studies; Table S4: GRADE Summary of Findings.

## Abbreviations

The following abbreviations are used in this manuscript:

CIs	Confidence Intervals
FBG	Fasting Blood Glucose
GRADE	Grading of Recommendations Assessment, Development and Evaluation
HbA1c	Glycated Hemoglobin A1c
HR	Heart Rate
LOO	Leave-one-out analysis
MD	Mean Difference
PRISMA	Preferred Reporting Items for Systematic Reviews and Meta-Analyses
RCTs	Randomised Controlled Trials
RoB 2	Cochrane Risk of Bias tool version 2
RoBANS	Revised Risk of Bias Assessment Tool for Nonrandomised Studies of Interventions
SD	Standard Deviation
SE	Standard Error
T2DM	Type 2 Diabetes Mellitus
