# Supplementary material for: Effects of Aquatic Exercise on Type 2 Diabetes Management in Adulthood: A Systematic Review and Meta-Analysis, Including Evidence on the Use of Wearable Devices
Source: Healthcare (Basel). 2026 Apr 10;14(8):998. doi: 10.3390/healthcare14080998 (PMC13115931; doi:10.3390/healthcare14080998)
Supplement: Supplementary file 1 [file healthcare-14-00998-s001.zip › Table S4.pdf]

Table S4: GRADE Summary of Findings

Outcome	Comparison (No. studies; n)	Risk of Bias	Inconsistency	Indirectness	Imprecision	Publication Bias	Certainty of Evidence	Effect Estimate MD (95% CI)
HbA1c (%)	Aquatic vs. Passive Control (8 studies listed; 7 contributing to meta-analysis; n = 157)	Serious [1]	Serious[2]	Not serious[3]	Not serious[4]	Not detected[5]	LOW	-0.76% (-1.21 to -0.32)
HbA1c (%)	Aquatic vs. Active Control (3 studies, n = 53)	Serious [1]	Not serious[6]	Not serious[3]	Serious[7]	Not assessable[8]	LOW	0.21% (-0.09 to 0.50)

[1] Risk of Bias (both subgroups): Downgraded one level. The majority of contributing studies rated as having some concerns under RoB 2 tool, primarily in the randomisation process and selection of reported results. One study [14] was rated as high risk of bias for HbA1c.
[2] Inconsistency (Aquatic vs. Passive Control): Downgraded one level. Considerable heterogeneity ($I^2 = 88\%$; $\tau^2 = 0.25$), with effects favouring both aquatic exercise and control conditions.
[3] Indirectness (both subgroups): Not downgraded. Populations, interventions, comparators, and outcomes directly relevant to the clinical question.
[4] Imprecision (Aquatic vs. Passive Control): Not downgraded. Confidence interval did not cross the null (95% CI -1.21 to -0.32) and excluded no effect.
[5] Publication Bias (Aquatic vs. Passive Control): Not downgraded. Funnel plot appeared broadly symmetrical; limited power with a modest number of studies.
[6] Inconsistency (Aquatic vs. Active Control): Not downgraded. No statistical heterogeneity observed ($I^2 = 0\%$; $p = 0.38$).
[7] Imprecision (Aquatic vs. Active Control): Downgraded one level. Three studies; n = 53, 95% CI crossed the null (-0.09 to +0.50); $p = 0.167$.
[8] Publication bias (Aquatic vs. Active Control): Not assessable. Insufficient number of studies (n = 3).
