# Supplementary material for: Effects of Aquatic Exercise on Type 2 Diabetes Management in Adulthood: A Systematic Review and Meta-Analysis, Including Evidence on the Use of Wearable Devices
Source: Healthcare (Basel). 2026 Apr 10;14(8):998. doi: 10.3390/healthcare14080998 (PMC13115931; doi:10.3390/healthcare14080998)
Supplement: Supplementary file 1 [file healthcare-14-00998-s001.zip › Table S1.pdf]

Supplementary Material

Table S1: Search queries used in different databases

**PubMed**	("Type 2 Diabetes Mellitus" OR "Type 2 Diabetes" OR "Diabetes Mellitus Type 2" OR T2DM OR NIDDM) AND (aquatic exercise* OR "aquatic aerobics" OR "aquatic training" OR "aquatic physical therapy" OR "aquatic rehabilitation" OR "water-based exercise*" OR "water exercise*" OR "water aerobics" OR "water therapy" OR swimming OR "in-water exercise") AND ("Glycated Hemoglobin" OR "Hemoglobin A1c" OR HbA1c OR A1c OR "Glycaemic Control" OR "Glycemic Control" OR "Fasting Blood Glucose" OR "Blood Glucose")
**Scopus**	ALL ("Type 2 Diabetes Mellitus" OR "Type 2 Diabetes" OR "Diabetes Mellitus Type 2" OR T2DM OR NIDDM) AND ALL ("aquatic exercise*" OR "aquatic aerobics" OR "aquatic training" OR "aquatic physical therapy" OR "aquatic rehabilitation" OR "water-based exercise*" OR "water exercise*" OR "water aerobics" OR "water therapy" OR swimming OR "in-water exercise") AND ALL ("Glycated Hemoglobin" OR "Hemoglobin A1c" OR HbA1c OR A1c OR "Glycaemic Control" OR "Glycemic Control" OR "Fasting Blood Glucose" OR "Blood Glucose")
**Web of Science**	TS=(("Type 2 Diabetes Mellitus" OR "Type 2 Diabetes" OR "Diabetes Mellitus Type 2" OR T2DM OR NIDDM) AND ("aquatic exercise*" OR "aquatic aerobics" OR "aquatic training" OR "aquatic physical therapy" OR "aquatic rehabilitation" OR "water-based exercise*" OR "water exercise*" OR "water aerobics" OR "water therapy" OR swimming OR "in-water exercise") AND ("Glycated Hemoglobin" OR "Hemoglobin A1c" OR HbA1c OR A1c OR "Glycaemic Control" OR "Glycemic Control" OR "Fasting Blood Glucose" OR "Blood Glucose"))
**Cochrane CENTRAL**	("Type 2 Diabetes Mellitus" OR "Type 2 Diabetes" OR "Diabetes Mellitus Type 2" OR T2DM OR NIDDM) AND ("aquatic exercise*" OR "aquatic aerobics" OR "aquatic training" OR "aquatic physical therapy" OR "aquatic rehabilitation" OR "water-based exercise*" OR "water exercise*" OR "water aerobics" OR "water therapy" OR swimming OR "in-water exercise") AND ("Glycated Hemoglobin" OR "Hemoglobin A1c" OR HbA1c OR A1c OR "Glycaemic Control" OR "Glycemic Control" OR "Fasting Blood Glucose" OR "Blood Glucose")
**IEEE Xplore**	("Type 2 Diabetes Mellitus" OR "Type 2 Diabetes" OR "Diabetes Mellitus Type 2" OR T2DM OR NIDDM) AND ("aquatic exercise" OR "aquatic aerobics" OR "aquatic training" OR "aquatic physical therapy" OR "aquatic rehabilitation" OR "water-based exercise" OR "water exercise" OR "water aerobics" OR "water therapy" OR swimming OR "in-water exercise") AND ("Glycated Hemoglobin" OR "Hemoglobin A1c" OR HbA1c OR A1c OR "Glycaemic Control" OR "Glycemic Control" OR "Fasting Blood Glucose" OR "Blood Glucose")
