# Supplementary material for: Effects of Aquatic Exercise on Type 2 Diabetes Management in Adulthood: A Systematic Review and Meta-Analysis, Including Evidence on the Use of Wearable Devices
Source: Healthcare (Basel). 2026 Apr 10;14(8):998. doi: 10.3390/healthcare14080998 (PMC13115931; doi:10.3390/healthcare14080998)
Supplement: Supplementary file 1 [file healthcare-14-00998-s001.zip › Table S2.pdf]

Table S2: Characteristics of included studies

Study	Populations					Intervention Duration	
Author, Year (Country)	n= (Sex)	Aquatic intervention (*n*=; Type)	Comparator group (*n*=; characteristics)	Age	Characteristics (e.g. other diseases, medication, …)	Duration, Frequency	Session duration (minutes)
Åsa et al., 2012 (Sweden) [31]	20 (4F|16M)	10; Aerobic	10; Standard care (passive)	65.8 ± 5.8 y	Patients with stable CHF and T2DM in NYHA class II or III, ejection fraction (EF) <50%	8 weeks, 3x/week	45 min
Nuttamonwarakul et al., 2012 (Thailand) [32]	40 (N.R)	20; Aerobic	20; Standard care (passive)	> 60 y	--	12 weeks, 3x/week	50 min
Nuttamonwarakul et al., 2014 (Thailand) [36]	19 (19F|0M)	10; Aerobic	9; Land-based exercise (active)	60-70 y	Participants were medically screened before participation to ensure that they were not altering medications during the exercise training period. (medication name not specified)	12 weeks, 3x/week	50 min
Delevatti et al., 2016 (Brazil) [39]	21(N.R)	11; Aerobic	10; Land-based exercise (active)	54.2 ± 8.3 y	--	12 weeks, 3x/week	45 min
Suntraluck et al., 2017 (Thailand) [37]	29 (N.R)	15; Aerobic	14; Land-base exercise (active)			12 weeks, 3x/week	Week 1-4: 35 min

Study	Wearable technologies	Outcomes				
Author, Year (Country)		Aquatic intervention HbA1c (Before; After)	Comparator group HbA1c (Before; After)	Aquatic intervention FBG (Before; After)	Comparator group FBG (Before; After)	Other
Åsa et al., 2012 (Sweden) [31]	--	7.9 ± 2.9; 7.2 ± 0.9 (%)	6.9 ± 2.0; 6.7 ± 3.2 (%)	10.2 ± 2.9; 9.3 ± 26 (mmol/L)	7.8 ± 3.3; 6.9 ± 2.0 (mmol/L)	VO_2max; blood lipid markers; scores of SF-36
Nuttamonwarakul et al., 2012 (Thailand) [32]	Heart rate monitor (Polar team 2pro, Finland)	7.7 ± 1.1; 6.6±0.7 (%)	7.6 ± 0.5; 7.6 ± 0.5 (%)	--	--	VO_2max; blood lipid markers; Cardiovascular function
Nuttamonwarakul et al., 2014 (Thailand) [36]	Heart rate monitor (Polar team 2pro, Finland)	8.1 ± 1.4; 6.7 ± 0.8 (%)	7.7 ± 0.5; 6.8 ± 0.3 (%)	146.2 ± 37.2; 135.6 ± 16.3 (%)	146.2 ± 37.2; 140.9 ± 35.6 (%)	CRP; insulin; MDA
Delevatti et al., 2016 (Brazil) [39]	Heart rate monitor (Polar, RSX 300, Kajaani, Finland)	7.42 ± 0.79; 7.00 ± 0.45 (%)	7.0 ± 0.45; 6.65 ± 0.38 (%)	169.8 ± 29.3; 160.1 ± 17.0 (mg/dl)	156.5 ± 16.6; 134.6 ± 7.7 (mg/dl)	VO_2max; blood lipid markers; Cardiovascular function; CRP
Suntraluck et al., 2017 (Thailand) [37]	Heart rate monitor (Polar FT7,	62.4 ± 3.5; 55.4 ± 2.3 (mmol/L)	63.2 ± 2.8; 55.5 ± 2.5 (mmol/L)	--	--	VO_2max; blood lipid markers;

	60-75 y	Participants had a baseline HbA1c value of 7–9% and were sedentary (no regular exercise training) in the past 6 months.Subjects were free of diabetic nephropathy, diabetic retinopathy, severe diabetic neuropathy, and cardiovascular and cerebrovascular diseases. Although all the subjects were on antihyperglycemic medications, none of the subjects changed the kind and dosage of medications throughout the investigation.			Week 5-8: 55 min<br><br>Week 9-12: 85 min	Kempele, Finland)					Cardiovascular function; body composition; leg strength: HOMA-IR
Conners et al.,2019 (USA) [40]	26 (16F|10M)	13; Aerobic	13; Standard care (passive)	12 weeks, 3x/week	30-60 min	Heart rate monitor (Polar, Finland)	7.25; Δ pre/post – 0.67 (%)	7.90; Δ pre/post – 0.02 (%)	--	--	Cardiovascular function; body composition; leg strength; cardiorespiratory fitness
	58.0 ± 5.0 y	T2DM for a minimum of 2 years									
Shourabi et al., 2020 (Iran) [38]	39 (N.R)	10; Resistance	9; Standard care (passive)	8 weeks, 3x/week	60 min	Heart rate monitor (Electro, Oy, Kempele, Finland)	HbA1c [-0.7% (95% CI, 0.9 to 0.6)		FBS [-18.1 mg/dl (95% CI, 22.4 to 13.7)		Insulin; Nerve growth factor; balance ability
	49.8 ± 2.3 y	Type 2 diabetic patients with peripheral neuropathy, that are able to walk a 1.6-km distance.<br><br>Medicated with metformin (500 mg, 2 times per day with meals) and glibenclamide (5–10 mg in the morning in a fasted state)									
Scheer et al., 2020 (Australia) [15]	27 (12F|15M)	13; Aerobic	14; Standard care (passive)	8 weeks, 3x/week	60 min	--	7.33 ± 0.97; 7.12 ± 1.07 (%)	6.88 ± 0.72; 7.04 ± 0.82 (%)	6.87 ± 0.93; 7.23 ± 1.21 (mmol·L−1)	8.18 ± 2.09; 8.46 ± 2.09 (mmol·L−1)	VO_2max; blood lipid markers; Cardiovascular function; body composition;
	60.9 ± 9.6 y	--									
Salarinia et al., 2023 (Iran) [33]	48 (48F|0M)	10; Resistance	10; Standard care (passive)	8 weeks, 3x/week	60 min	--	8.57 ± 1.5; 7.95 ± 1.34 (%)	7.39 ± 1.18; 7.3 ± 1.11 (%)	138.55 ± 63.54; 130.66 ± 17.57 (mg/dl)	114.28 ± 54.14; 110 ± 11.34 (mg/dl)	VO_2max; blood lipid markers; Cardiovascular function; body composition; HOMA-IR
	41.5 ± 2.7 y	--									
Polydang et al., 2023 (Thailand) [34]	33 (21F|12M)	16; Aerobic	17; Standard care (passive)	12 weeks, 3x/week	60 min	Heart rate monitor (Polar H10, Kempele, Finland)	7.8 ± 0.7; 7.0 ± 0.7 (%)	7.9 ± 0.7; 7.6 ± 0.6 (%)	135 ± 21; 117 ± 22 (mg/dl)	147 ± 25; 152 ± 40 (mg/dl)	Cardiovascular function; body composition; cognitive function;
	68.9 ± 3.7 y	Participants had a baseline HbA1c value of 7%–9%. Participants had mild cognitive impairment (MCI) without diabetes complication issues (e.g. neuropathy) not treated with insulin injection.									

Bonab et al., 2023 (Iran) [35]	60 (60F|0M)	20; Aerobic and Resistance	20; Standard care (passive)	12 weeks, 3x/week	60 min	Heart rate monitor (Polar, Finland)	7.88 ± 0.12; 6.12 ± 0.14 (%)	5.41 ± 0.22; N.R (%)	172.96 ± 0.70; 143.76 ± 4.87 (mg/dl)	88.12 ± 0.77; n.a. (mg/dl)	Lipocalins
	54.02 ± 12.03 y	No chronic illnesses and no diabetes complications									

Note: all included studies were RCT. Abbreviations: F: female; M: male; N.R: not reported; CHF: Chronic heart failure; T2DM: Type 2 diabetes mellitus; CRP - C-reactive protein; FBS - Fasting Blood Glucose; MDA - malondialdehyde; CRF - cardiorespiratory fitness
