# Supplementary material for: Effects of Aquatic Exercise on Type 2 Diabetes Management in Adulthood: A Systematic Review and Meta-Analysis, Including Evidence on the Use of Wearable Devices
Source: Healthcare (Basel). 2026 Apr 10;14(8):998. doi: 10.3390/healthcare14080998 (PMC13115931; doi:10.3390/healthcare14080998)
Supplement: Supplementary file 1 [file healthcare-14-00998-s001.zip › Table S3.pdf]

Table S3: Summary of HbA1c outcomes across included studies

Study	Intervention (n)	Control (n)	HbA1c baseline Intervention Mean ± SD (%)	HbA1c post Intervention Mean ± SD (%)	HbA1c baseline Control Mean ± SD (%)	HbA1c post Control Mean ± SD (%)	Type of control (Passive/Active)
Åsa et al., 2012 [31]	10	10	7.9 ± 2.9	7.2 ± 0.9	6.9 ± 2.0	6.7 ± 3.2	Passive
Nuttamonwarakul et al., 2012 [32]	20	20	7.7 ± 1.1	6.6 ± 0.7	7.6 ± 0.5	7.6 ± 0.5	Passive
Nuttamonwarakul et al., 2014 [36]	10	9	8.1 ± 1.4	6.7 ± 0.8	7.7 ± 0.5	6.8 ± 0.3	Active
Delevatti et al., 2016 [39]	11	10	7.42 ± 0.79	7.00 ± 0.45	7.00 ± 0.45	6.65 ± 0.38	Active
Suntraluck et al., 2017 [37]	15	14	7.86 ± 2.47	7.22 ± 2.36	7.93 ± 2.41	7.23 ± 2.38	Active
Conners et al., 2019 [40]	13	13	7.25 ± 0.67	6.58 ± 0.40	7.90 ± 0.02	7.92 ± 0.08	Passive
Scheer et al., 2020 [15]	13	14	7.33 ± 0.97	7.12 ± 1.07	6.88 ± 0.72	7.04 ± 0.82	Passive
Salarinia et al., 2023 [33]	10	10	8.57 ± 1.50	7.95 ± 1.34	7.39 ± 1.18	7.30 ± 1.11	Passive
Ploydang et al., 2023 [34]	16	17	7.8 ± 0.7	7.0 ± 0.7	7.9 ± 0.7	7.6 ± 0.6	Passive
Bonab et al., 2023 [35]	20	20	7.88 ± 0.12	6.12 ± 0.14	7.86 ± 0.13	7.70 ± 0.28	Passive

Shourabi et al., (2020) [38] did not report complete HbA1c data and was therefore not included in the meta-analysis
